# Suboptimal Temperature Acclimation Affects Kennedy Pathway Gene Expression, Lipidome and Metabolite Profile of *Nannochloropsis salina* during PUFA Enriched TAG Synthesis

**DOI:** 10.3390/md16110425

**Published:** 2018-11-01

**Authors:** Saba Shahid Gill, Stephanie Willette, Barry Dungan, Jacqueline M. Jarvis, Tanner Schaub, Dawn M. VanLeeuwen, Rolston St. Hilaire, F. Omar Holguin

**Affiliations:** 1 Department of Plant and Environmental Sciences, New Mexico State University, Las Cruces, NM 88003, USA; sabagill@nmsu.edu (S.S.G.); stephwi@nmsu.edu (S.W.); bdungan@nmsu.edu (B.D.); jmjarvis@nmsu.edu (J.M.J.); rsthilai@nmsu.edu (R.S.H.); 2 Chemical Analysis and Instrumentation Laboratory, New Mexico State University, Las Cruces, NM 88003, USA; tschaub@nmsu.edu; 3 Department of Economics, Applied Statistics and International Business, New Mexico State University, Las Cruces, NM 88003, USA; vanleeuw@nmsu.edu

**Keywords:** *Nannochloropsis salina*, cold stress, fatty acid methyl ester (FAME) analysis, polyunsaturated fatty acids (PUFA), eicosapentanoic acid (EPA), Kennedy pathway, lipidomics, metabolomics, lipid remodeling

## Abstract

In humans, dietary polyunsaturated fatty acids (PUFAs) are involved in therapeutic processes such as prevention and treatment of cardiovascular diseases, neuropsychiatric disorders, and dementia. We examined the physiology, PUFA accumulation and glycerol lipid biosynthesis in the marine microalga *Nannochloropsis salina* in response to constant suboptimal temperature (<20 °C). As expected, *N. salina* exhibited significantly reduced growth rate and photosynthetic activity compared to optimal cultivation temperature. Total fatty acid contents were not significantly elevated at reduced temperatures. Cultures grown at 5 °C had the highest quantity of eicosapentanoic acid (EPA) (C20:5n3) and the lowest growth rate. Additionally, we monitored broadband lipid composition to model the occurrence of metabolic alteration and remodeling for various lipid pools. We focused on triacylglycerol (TAG) with elevated PUFA content. TAGs with EPA at all three acyl positions were higher at a cultivation temperature of 15 °C. Furthermore, monogalactosyldiacylglycerol and digalactosyldiacylglycerol, which are polar lipids associated with chloroplast membranes, decreased with reduced cultivation temperatures. Moreover, gene expression analysis of key genes involved in Kennedy pathway for de novo TAG biosynthesis revealed bimodal variations in transcript level amongst the temperature treatments. Collectively, these results show that *Nannochloropsis salina* is a promising source of PUFA containing lipids.

## 1. Introduction

In humans, deficiency of long chain polyunsaturated fatty acids (PUFAs) increases the risk of cardiovascular disease, hypertension, inflammatory disease, and neuropsychiatric disorders, such as depression and dementia [1,2]. For the prevention of coronary heart disease and neural disorders, dietary recommendations include consumption of 1 g PUFAs per day by the American Heart Association and the European Society of Cardiology [3].

Fish and fish oils are the most common human dietary sources of PUFAs. The long chain omega-3–PUFA (omega-3–polyunsaturated fatty acid 20:5) and DHA (docosahexanoic acid 22:6n-3) are essential for cell membranes and found in high proportion in external segments of photoreceptors and neuronal membranes [4,5]. Furthermore, due to the number of double bonds in PUFAs, they act as antioxidants scavenging reactive oxygen species (ROS) [6,7]. Omega-3 and omega-6 fatty acids are considered to be heart healthy and are beneficial to patients suffering from cognitive disorders [4].

Microalgae are an alternative source for consumer PUFA production [8]. Microalgae produce and accumulate a variety of biochemical compounds from diverse metabolic pathways, which allows them to survive in highly competitive environments. Natural products of microalgae include carotenoids, fatty acids (FA), polyketides, mycosporine-like amino acids, lectins, phycocolloids, and toxins [9,10]. The ability of unicellular algae to synthesize a diversity of compounds, higher biomass productivity, and relatively high growth rate make microalgae a strong candidate as feedstock for high value products at the industrial level [11,12]. Exploitation of microalgae has opened new prospects for research and development within several industries, including pharmaceuticals, nutraceuticals, and biofuels [13,14].

Lipid accumulation in microalgae occurs mainly in three forms; triacylglycerides (TAGs), saturated fatty acids, and PUFAs [15]. TAGs are the main form of lipid accumulation and primary storage molecules for carbon and energy [16,17]. Growth temperature affects the distribution of various lipid pools in the cells [15,18,19]. Previous work has shown that exposure to a broad range of temperatures results in fluctuations in the proportion of saturated and unsaturated fatty acids for membrane lipids, which contributes to cellular acclimation and photosynthetic thermo-stability [20,21]. Other than cold stress, several abiotic factors are directly involved in lipid accumulation in microalgae such as nitrogen deprivation, salinity, osmotic pressure, heat [22], chemicals, and drugs [23,24].

The Kennedy pathway is involved in de novo synthesis of TAGs; its reactions consist of stepwise acylation, adding to each hydroxyl group of glycerol beginning with glycerol-3-phosphate [25]. After synthesis, TAGs are accumulated in lipid bodies, which are single-layer, membrane-wrapped, protein embedded organelles that are 0.2–2.5 μm in diameter that consist of ~90% TAGs and 10% free fatty acids. These lipid bodies are present in the cytoplasm of nearly all plant and algal cells [26,27]. It is generally believed that plant lipid bodies are not only a cellular lipid reservoir, but also provide energy during seed germination. Microlagal lipid accumulation can be in response to maintaining cellular redox homeostasis, and to high concentrations of ATP, NADH, and acetyl-CoA [28].

Here, we selected *Nannochloropsis salina* (CCMP 1776), a marine microalga found in the Eustigmatophyceae class, for its ability to grow in a high saline environment (e.g., brackish water) and for the demonstrated PUFA accumulation of this species [29]. We define the effect of continuous, reduced cultivation temperature on physiological parameters, metabolic pool alteration, lipid metabolism, and Kennedy pathway gene expression to understand the mechanism of EPA incorporation in triglycerides.

## 2. Results

### 2.1. Culture Acclimation and Cell Number

Cold stress or suboptimal temperature is a major trigger for plastic metabolism of lipids in microalgae, as plants and green algae acclimate to cold environment by changing their metabolic pathways [30]. Information about the metabolic pathways that govern the behavior of microalgae under cold stress is scant [31]. Therefore, it is necessary to define cold stress for each unique strain of microalgae due to their different responses under low temperature cultivation condition. This study was performed to bridge the gap in understanding cold stress responses in *Nannochloropsis salina* CCMP1776.

Analyses of the various growth parameters were performed to define cold stress in *N. salina* and to verify the relationship between cold stress and reduced growth. *Nannochloropsis salina* CCMP1776 showed distinct reduction in physiological responses such as growth rate and cell number. Cells of *N. salina* were cultivated in F/2 media at four different temperatures (25, 15, 10, and 5 °C) in ePBRs. Cell cultures were inoculated at an initial optical density (OD_750_ nm) of 0.44–0.46. After an acclimation period of 48 h, the cultivation temperatures were reduced and the cultures were acclimated to their respective suboptimal temperatures. After 96 h of reduced temperature treatment, the OD_750_ nm was 0.60 for 25 °C, 0.50 for 15 °C, 0.51 for 10 °C, and 0.49 for 5 °C (Table 1). The optical density of the culture grown at 5 °C is 17.5% lower than those grown at 25 °C. The results indicate that acclimated algal cells show significant differences in OD_750_ nm of *N. salina* at reduced temperatures (0.52 ± 0.02 on 72 h at 15 °C as compared to 0.49 ± 0.02 on 48 h at 10 °C). We have calculated growth rates (day^−1^) for *N. salina* which were 0.08, 0.06, 0.05, and 0.04 for 25, 15, 10, and 5 °C respectively (Table S2).

Significant differences in total cell count between cultures acclimated at different temperatures during the 96 h growth period are observed (Table 1). For total cell count determined by light microscopy, the 2 mL sample drawn from each culture is assumed to represent the cellular population. The cell count of 1.40 × 10^10^ for 25 °C was significantly higher as compared to 1.2 × 10^9^ for 15 °C, 9.5 × 10^9^ for 10 °C and 7.82 × 10^9^ for 5 °C after the 96 h growth period (Table 1). A two-way measured ANOVA was performed to analyze significance between two factors, temperature and growth period. After 96 h, cultures grown at 25 °C had 44.1% (*p* < 0.05) more cells as compared to those grown at 5 °C (Table 1). However, there were minor differences in the cell count during 48 h of growth period (7.2 × 10^9^ ± 2.4 × 10^8^ at 15 °C as compared to 6.9 × 10^9^ ± 2.4 × 10^8^ at 10 °C). These results indicate that suboptimal temperature treatment hinders normal algal cell division in the cultures, while redirecting cellular metabolism towards energy storage.

### 2.2. PAM Fluorometery

We evaluated photosynthetic efficiency of algal cells by measuring maximal quantum yield of PSII (Fv/Fm) using PAM (Pulse-Amplitude-Modulation) fluorometer. Fv/Fm ratio was measured at all four reduced temperatures every 24 h. Post inoculation Fv/Fm values ranged from 0.54–0.55. Over the course of 96 h, the Fv/Fm values gradually decreased from 0.55 for 25 °C to 0.50, 0.39, and 0.28 for suboptimal temperature treatments 15, 10, and 5 °C respectively (Table 1, Fv/Fm). Decreased Fv/Fm values were correlated with reduced temperatures. Significant differences appeared after 24 h at 5 °C in contrast to 10, 15, and 25 °C (Table 1). Reduced temperature affects the photosynthetic activity by inhibiting chlorophyll accumulation and reducing the utilization of light harvesting complex. Photosynthetic activity and electron transport rates were found to be decreased at reduced cultivation temperatures [32]. Electron transport rate measured at 280 µmol photons m^−2^·s^−1^, decreased significantly at 5 °C (5.50 µmol photons m^−2^·s^−1^) as compared to those held at 25 °C (15.80 µmol photons m^−2^·s^−1^) (Table 1). Maxwell showed that photosynthetic activity was reduced by 25% for *Chlorella* during cold stress [33], which is similar to our finding for *N. salina*. Photosynthetic activity and de novo fatty acid synthesis are both dynamic, energetically demanding reactions that occur within algal cells. A marked decrease in photosynthetic activity suggests a shift in energy appropriation and carbon assimilation within the cell. Taken together, cell number and PAM fluorometery data suggest that cold stress (15–5 °C) significantly lowers the growth rate and photosynthetic activity of the *N. salina* cell.

### 2.3. Dissolved Oxygen Measurements

Dissolved oxygen (DO) is a direct measure of photosynthetic activity in aquacultures, and is produced as a byproduct during photosynthesis and taken up by the cells as energy source to break glucose molecule during respiration at night. The data collected were corrected for temperature, (atmospheric pressure (640 mmHg) and salinity (3.5%) [34]. After the 48-h acclimation period, statistically significant different DO values (119.04% at 25 °C, 86.37% at 15 °C, 63.88% at 10 °C, and 70.76% at 5 °C) were observed at 0 h of reduced temperature treatment, (Table 1). We observed post acclimation adjustments in DO (88.99% at 15 °C and 86.75% 10 °C) after 24 h of growth. At 96 h, % saturation of oxygen was 93.29% for cultures grown at 25 °C, 88.48% at 15 °C, 89.50% at 10 °C and 69.85% at 5 °C, which indicate a decrease in photosynthesis at reduced temperatures. Although all the temperatures show a significant difference in % saturation of oxygen during the 96 h growth period, the culture at 5 °C shows a drastic decrease in % saturation of oxygen. A previous study demonstrated a similar effects of suboptimal temperatures and dissolved oxygen on photosynthetic activity (Fv/Fm) [35]. However, the role of cellular respiration during night cycle cannot be ignored [36]. These findings indicate that, the 5 °C temperature exerts more stress on algal cells and halts its efficiency to perform photosynthesis accompanied with reduced % saturation of oxygen, which is crucial for survival. Muhlroth et al. indicated that in the presence of oxygen inside the cell, saturated fatty acids are converted into unsaturated fatty acids by enhancing enzymatic activity of desaturases [37]. DO measurement gives us an insight into oxygen levels in the culture. As the study of cellular level of oxygen was not performed for this experiment, these results suggest that a future study of the cellular oxygen level will help elucidate the effect of temperature on inner cellular environment.

### 2.4. Elemental Analysis

Major elements of organic substance (carbon, hydrogen, nitrogen, oxygen, and sulfur), which are precursors for lipids, protein, and carbohydrates in algal cells, are determined by combustion analysis. The elemental analysis revealed that there were only subtle differences between temperature treatments on carbon and nitrogen contents. Carbon contents were different for 15 °C (44.1% ± 0.4) relative to other temperature treatments (41.3% ± 0.8 for 25 °C, 41.9% ± 0.4 for 10 °C and 40.9% ± 1.5 for 5 °C) with a similar trend for nitrogen (7.0% ± 0.1 for 15 °C as compared to 7.0% ± 0.1 for 25 °C, 6.0% ± 0.1 for 10 °C, and 5.8% ± 0.2 for 5 °C) contents over temperature treatments. After 96 h of growth, there are statistically non-significant differences between temperature treatments for C/N at 15 °C (6.7 for 15 °C, as compared to 6.9 for 25 °C, 7.0 for 10 °C, 7.0 for 5 °C) (Table 2). Total protein content of algal biomass has a convincing impact in determining the fluctuation of cellular activities under stress. We noticed non-significant differences in total protein content determined by % N content at suboptimal temperature treatment 15 °C (31.5%) compared to control and other temperature treatment (~28.5%). This increase in total estimated protein content at low temperature might be associated with carbohydrate metabolism and lipid biosynthesis as can be seen in fold changes in the primary lipid pool (Table 4).

### 2.5. Fatty Acid Methyl Ester (FAME) Analysis

Fatty acids have a significant role in the survival of a cell under stress conditions. Their role in membrane fluidity, permeability, integrity, and as energy storage bodies makes them vital components of algal cells. The ratio of saturated and unsaturated fatty acids changes in membranes in suboptimal growth conditions, where the stress response mechanism of the cell may be elucidated. Fatty acid methyl ester (FAME) analysis was performed to determine the cellular response of cultures grown at 25, 15, 10, and 5 °C at a growth period of 96 h. Total fatty acid (TFA) concentration did not change significantly with temperature but subtle differences were detected. Total fatty acid content was 16.2% higher for cultures grown at 5 °C relative to optimum temperature. The ratio of total FAME to total biomass remained unchanged with decreased temperature (Table 3), which indicates that suboptimal growth temperature increases lipid content at the cost of biomass productivity, while lipid productivity remained unchanged [38]. The abundance of the saturated fatty acids C14:0, C16:0, and C18:0 and their unsaturated counterparts did not change with growth temperature. The concentration of mono and di-unsaturated fatty acids also remained consistent with growth temperature. Eicosastrienoic acid (C20:3n6) showed a significant increase at 5 °C and 10 °C (Table 3). Eicosapentanoic acid content only changed minimally with the highest values observed at 15 °C growth temperature. Although differences in the distribution of FAME components were detected in response to reduced temperature, eicosatetraenoic acid (C20:4n6) content was not statistically different among the treatment temperatures. Production of omega-3 fatty acid (C20:5n3) was 1.2-fold higher for 5 °C samples than the control temperature whereas a slight decrease in EPA accumulation was observed at 10 °C and 15 °C. Total saturated fatty acids (SFA) concentrations were not statistically different for samples of variable growth temperature. The concentration of PUFAs was 21% more for 5 °C, while 15 °C showed a 3.2% decrease in PUFA as compared to control temperature.

The fatty acid profile of *Nannochloropsis salina* showed that saturated fatty acid to TFA, unsaturated to TFA, and PUFA to TFA ratios were significantly different (*p* < 0.05) for suboptimal temperatures (15, 10, and 5 °C) compared to the control. FAME analysis indicated that the fatty acid distribution on glycerol lipids differs in *N. salina* with cultivation temperature. These findings are consistent with literature where lipid content was shown to increase with slight decreases in cultivation temperature [38]. Cultivation of *Nannochloropsis* sp. at low temperature yields more EPA and PUFAs in the total lipid pool [37]. Similarly, the proportion of both total and target FAMEs increases with decreasing temperature (15 °C, 10 °C); however, for these two treatments, EPA proportion was correspondingly reduced. The EPA precursor C20:3n6 was more abundant at suboptimal temperatures than in optimally grown cultures. The highest TFA contents were produced at 5 °C. Interestingly, the 5 °C treatment resulted in both a higher EPA and EPA precursor content than the control and other temperature treatments. This result suggests that 5 °C is near-optimal for stimulation of EPA accumulation on a dry mass per day production basis.

### 2.6. Gene Expression Analysis of Four Kennedy Pathway Genes

**Figure 1:**
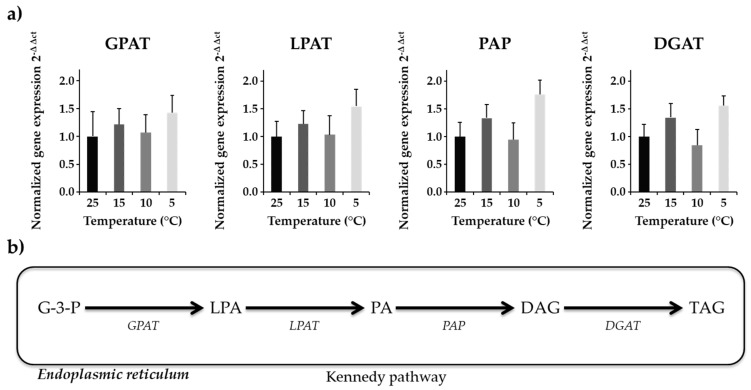
Gene expression analysis of four Kennedy pathway enzymes (**a**) Normalized gene expression (2^−ΔΔ*C*t^) of four genes with beta actin as internal control is shown. The graph represents averaged values (*n* = 12) along with standard error means in standard error bars. (**b**) Schematic representation of the Kennedy pathway. These results showed bimodal variation in gene expression level at different temperature.

Gene expression analysis is a key to understand the transcriptional alterations and metabolic pathway regulations of cellular activity under environmental changes. In *N. salina*, highly polyunsaturated fatty acids accumulate under cold stress [39]. Four enzymes (glycerol-3-phosphate acyltransferase—GPAT, lysophosphatidyl-choline acyltransferase—LPAT, phosphatidic acid phosphatase—PAP, diacylglycerol acyltransferase—DGAT) involved in Kennedy pathway for de novo biosynthesis of TAGs were studied to analyze differences in their transcript level under reduced temperature treatment. RNA was harvested from the cells 8 h into the light cycle, when TAG biosynthesis was favored under stress. The transcript abundance of genes involved in the acyl dependent synthesis of TAGs was evaluated. Normalized gene expression (2^−ΔΔ*C*t^) showed a bimodal trend of gene expression over temperature treatment (Figure 1).

Expression of GPAT transcript, which is a very critical enzyme in TAG biosynthesis, showed non-significant differences among treatment groups. Average expression of GPAT was slightly upregulated for 15 °C and 5 °C as compared to 10 °C and 25 °C treatments (Figure 1). Gene expression of LPAT, which adds second acyl chain to the product of GPAT, showed similar results as with the GPAT enzyme. Although expression of LPAT was upregulated for 15 °C and 5 °C as compared to 10 °C and 25 °C (Figure 1), the results were non-significant. Approximately a two-fold increase in PAP transcript level at 5 °C relative to control temperature was observed (Figure 1). DGAT expression was upregulated (~1.5-fold higher) at 5 °C and 15 °C as compared to 25 °C (Figure 1). This upregulation in DGAT transcript level at 5 °C correlated with the TAG biosynthesis at corresponding temperature indicate that algal cells produce more TAGs at suboptimal temperatures (Table 4).

### 2.7. Lipid Composition

We utilize high resolution, accurate mass measurement FT-ICR mass spectrometry to monitor broadband lipid compositional change with growth temperature. Mass spectral signals are normalized to sample weight for each sample. In positive ionization mode, sodium acetate added to the electrospray solution enables the formation of sodium adducts ([M + Na]^+^) for the mass spectral observation of diacylglycerols (DAGs), triacylglycerols (TAGs), monogalactosyl monoacylglycerol (MGMG), mono- and di-galactosyl-diacylglycerols (MGDG, DGDG), and mono- and di-acylglycerol trimethyl homoserine (MGTS, DGTS). Sulfonated and phosphorylated lipid species including sulphoquinovosyl diacylglycerol (SQDG) and phosphotidyl diacylglycerol (PG) are detected as deprotonated negative ions. Sub-part per million mass measurement accuracy, Kendrick mass sorting and isotopic fine structure analysis enable elemental composition assignment, after which the number of acyl carbon atoms and double bonds ([Acyl-carbon:DBE]) is determined. Molecular double bond equivalents (DBE) are determined for each lipid elemental composition and the acyl carbon number is calculated by subtracting the glyceride part and respective head group from the total lipid species elemental composition. The acyl carbon number and DBE distribution of each lipid class and the relative abundance values for each class are calculated relative to total monoisotopic lipid signal. Summed relative abundances for the species within each lipid class are used to provide a qualitative view of lipid change among treatments and control samples.

**Figure 2:**
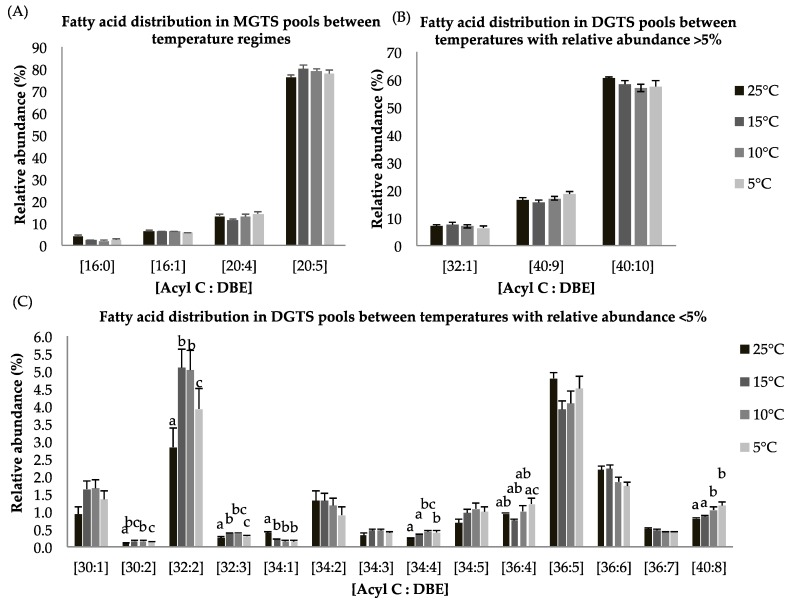
(**A**–**C**): Lipid acyl distributions for MGTS and DGTS. Fatty acid distribution of MGTS and DGTS lipid species across temperature on the basis of acyl carbon and unsaturation: Relative abundance of lipid species was calculated by averaging S/N values obtained by positive ionization mode from biological triplicates (*n* = 3); ±SEM are shown, where a, b, c, d represents statistically significant mean separations of differences at *p* ≤ 0.05.

Eight lipid classes (MGTS, DGTS, DAG, TAG, MGDG, DGDG, SQDG, and PG) among four temperature treatments were observed. The betaine lipids are comprised of both mono and diaclycglercol trimethyl-homoserine (MGTS, DGTS). The highest MGTS contents were observed at 15 °C (Table 4). MGTS (20:5), a lipid species containing one molecule of EPA (C20:5), accounts for >80% of the total MGTS signal observed for all temperatures (Figure 2A). DGTS content was slightly higher for 15 and 10 °C while no increase in betaine lipid was observed at 5 °C (Table 4).

Both, MGTS and DGTS had higher EPA contents (i.e., [C20:5], [C40:10], Figure 2A,B), which indicates that long chain polyunsaturated lipids are retained during temperature-dependent lipid alteration. Within the DGTS lipid pool, several lipid species showed statistically significant difference in response to reduced temperature treatment. The abundance of lipid species [C30:2], [C32:2], [C32:3], [34:4], [36:4], and [40:8] were higher at reduced temperature while the relative abundance of [34:1] was lower at suboptimal temperatures (Figure 2C). A previous study has shown that DGTS is used as a precursor of galactolipids (DGDG) and EPA-rich lipid molecules [40,41,42].

**Figure 3:**
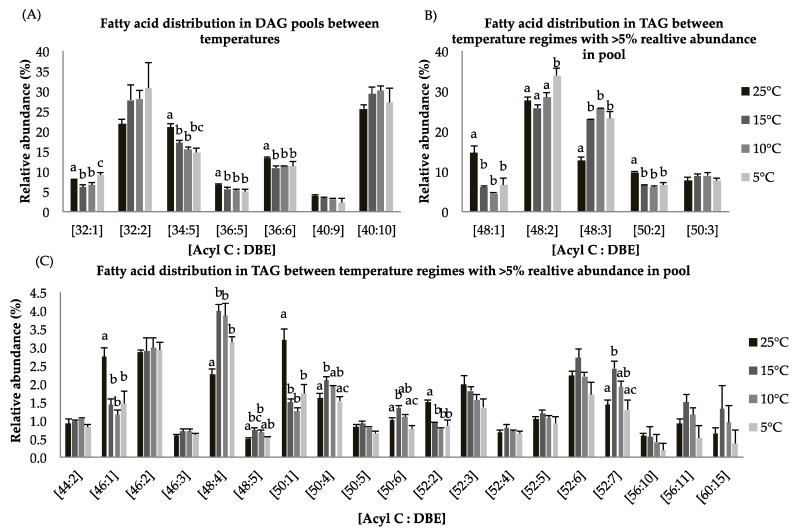
(**A**–**C**): Lipid acyl distributions for DAG and TAG. Fatty acid distribution of DAG and TAG lipid species across temperature on the basis of acyl carbon and unsaturation: Relative abundance of lipid species was calculated by averaging S/N values obtained by positive ionization mode from biological triplicates (*n* = 3); ±SEM are shown, where a, b, c, d represents statistically significant mean separations of differences at *p* ≤ 0.05.

**Figure 4:**
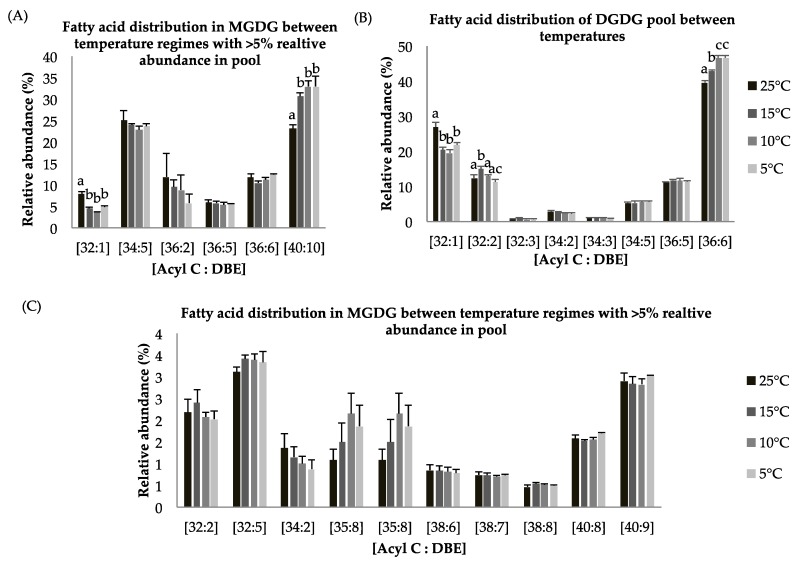
(**A**–**C**): Lipid acyl distributions for MGDG and DGDG. Fatty acid distribution of MGDG and DGDG lipid species across temperature on the basis of acyl carbon and unsaturation: Relative abundance of lipid species was calculated by averaging S/N values obtained by positive ionization mode from biological triplicates (*n* = 3); ±SEM are shown, where a, b, c, d represents statistically significant mean separations of differences at *p* ≤ 0.05.

Overall, the diacylglycerol (DAG) content was lower in the 5 °C cultures (1.7 folds) compared to the 25 °C cultures (Table 4). The fatty acid distribution within the DAG pool indicates that species [C32:1], [C34:5], [C36:5], and [C36:6] show a significant decrease in relative abundance at constant suboptimal temperatures while [C32:2] and [C40:10] increase (Figure 3A). TAG production was greater at 5 °C as compared to control temperature by 1.3 folds (Table 4). Based on gene expression data and lipid compositional analysis, we speculate that most DAG species incorporated into TAG were not direct products of de novo biosynthesis but result from lipid remodeling. For triyglycerides, long chain FA species [C48:1] show statistically significant decrease for cultures grown at suboptimal temperatures as compared to control temperature. About 35% of TAGs consist of the FA species [C48:2] at 5 °C whereas its contribution was <30% of total lipids at 25 °C (Figure 4B). Previous studies have shown that long chain polyunsaturated fatty acids (LC-PUFAs) are accumulated in more complex polar membrane lipids to increase membrane fluidity of the cell [43].

For triglycerides, the FA species [C46:1], [C48:1], [C48:5], [C50:1], [C50:4], [C50:6], [C52:2], and [C52:7] are statistically different with *p <* 0.05 at all temperature treatments (Figure 4C). Fatty acid species with less than 5% relative abundance contained the most important lipid species [C56:11] and [C60:15], which are EPA-rich TAG molecules. These species showed higher relative abundances at 15 °C relative to the other temperature treatments (Figure 4C). These findings indicate that incorporation of LC-PUFA into TAGs involves lipid remodeling under reduced temperature treatment. An alternative pathway to convert membrane lipids into TAGs involves phospholipid/diacylglycerol acyltransferase (PDAT). It has been shown that upregulation of the PDAT enzyme under nitrogen deprived culture conditions contributed to lipid remodeling [44].

Galactolipids monogalactosyl diacylgylcerol (MGDG) and digalactosyl diacylgylcerol (DGDG) lipid classes, which are an integral parts of thylakoid membranes, show statistically significant decreases at reduced growth temperature as compared to control temperature (*p <* 0.05, Table 4) which indicates degradation of membrane lipids and possible support for TAG biosynthesis through lipid remodeling. These findings complement photosynthetic measurement, which showed a decrease in Fv/Fm values at suboptimal temperature. The MGDG lipid pool has around 5% and 25% of the EPA-containing lipid species [C40:9] and [C40:10] (Figure 4A,C). The MGDG lipid pool shows a statistically significant increase in [C40:10] species at suboptimal temperature (Figure 4A). A similar pattern for the DGDG lipid pool was observed. DGDG is a product of the MGDG lipid pool after a second galactosylation and indicates this lipid class is involved in lipid remodeling under temperature stress. The MGDG pool consists of the [C40:10] fatty acid species in abundance (~25–35%) (Figure 4A), whereas the DGDG pool contains a relatively higher abundance (~45%) of the [C36:6] FA species (Figure 4B). Other detected lipid species including [C32:5], [C34:5], [C36:5], [C36:6] in the MGDG and [C32:3], [C34:2], [C34:3], [C34:5], [C36:5] in the DGDG lipid pools (Figure 4A–C) were observed and show non-significant differences in abundance among temperature treatments.

**Figure 5:**
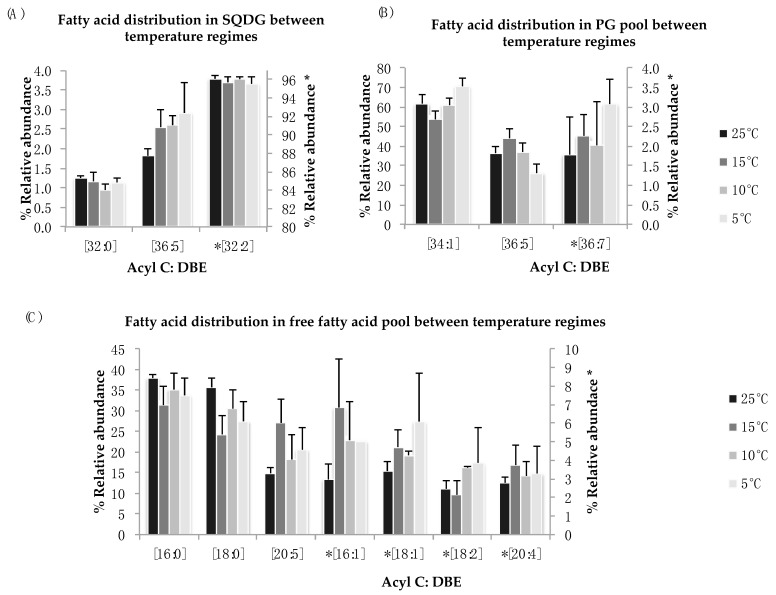
(**A**–**C**): Lipid acyl distributions for SQDG, PG, and FFA. Fatty acid distribution of SQDG, PG, and FFA lipid species across temperature on the basis of acyl carbon and unsaturation: Relative abundance of lipid specie was calculated by averaging S/N values obtained by positive ionization mode from biological triplicates (*n* = 3); ±SEM are shown (*p* ≤ 0.05). * Indicate secondary *y*-axis to show relative abundances.

Sulfoquinovosyl diacylglycerol (SQDG) and phosphatidyl-glycerol (PG) are a major part of chloroplastic membranes and are mainly involved in the structural integrity of membranes under stress condition. In *N. salina*, these two lipid classes showed no statistical differences in the distribution of fatty acid components for all temperature treatments (Figure 5A,B). A non-significant increase was noticed in FA species of SQDG [C36:5] and PG [C34:1] and [C36:7] for reduced temperature treatment. The most abundant polyunsaturated lipid species observed in SQDG lipid pool is [C32:2] (Figure 5A). Fluctuations in the SQDG [C36:5] PUFA species were observed. We observe an increase in mono-unsaturated lipid species [C34:1] in PG lipid pool at 5 °C and 10 °C (Figure 5B). Decrease in abundant lipid species [C34:1] is observed for suboptimal temperatures in PG lipid pool except at 15 °C, where the relative abundance of this FA species is higher as compared to 25 °C. For the low abundant PUFA species in PG lipid pool [C36:7], minor variances among treatment groups are observed.

The free fatty acid distribution across temperature treatments show non-significant fluctuations for palmitic acid [C16:0], stearic acid [C18:0], eicosapentanoic acid (EPA) [C20:5], and their mono and di-saturated derivatives (Figure 5C). The FFA distribution across suboptimal temperature provides insight into lipid degradation and remodeling [45].

### 2.8. Metabolite Analysis

**Figure 6:**
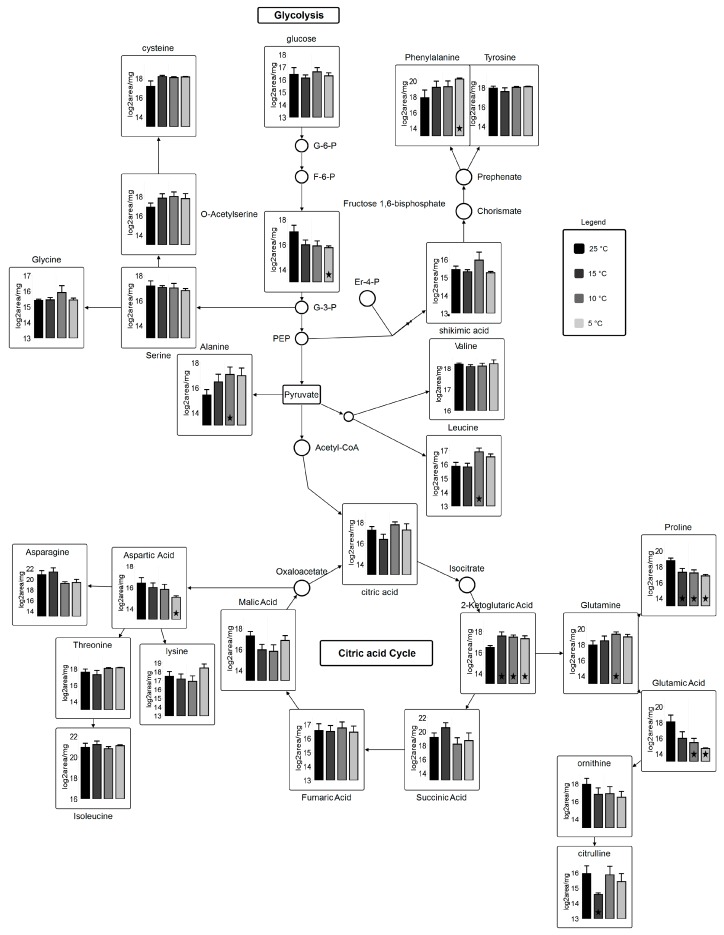
Metabolic pathway for carbohydrate metabolism coupled with amino acid biosynthesis. Circles represent undetected metabolites, averaged log_2_ transformed data of biological triplicates (*n* = 9) is shown in the graphs, VANTED software used to perform *t*-test to detect significant changes in metabolome. ★ represents statistically significant difference as compared to 25 °C (*p* ≤ 0.05).

Two hundred and six metabolite peaks were detected and deconvulated resulting in 69 metabolites identified by GC-TOF-MS using retention index and Fiehn number. Mass spectral peak intensities were normalized to sample weight and the data set was transformed by log2 for metabolomics data analysis. A number of classes were identified including sugars, sugars with phosphate groups, organic acids, fatty acids, amino acids and its derivatives, amides, polyamines, saccharides, and others. Carbohydrate metabolism, amino acid biosynthesis, and fatty acid biosynthesis pathway mapping was performed in VANTED using log2 transformed data (Figure 6).

Plant and photosynthetic organisms are continuously exposed to temperature stress and hence are subjected to changes in their metabolome [46,47]. Metabolite analysis revealed that 19 out of 69 analyzed metabolites show major and subtle alterations in the most abundant primary and secondary metabolites after the 96-h growth period across variant temperatures (Table S3). Sugars, which are involved in TCA cycle, are entailed in carbon skeleton allocation into fatty acids and other primary metabolites. Glucose metabolite pool, which is the most desired precursor molecule for a cell to carry out different reactions, is decreased at suboptimal temperatures as compared to control. Most importantly, 2-Ketoglutaric acid shows statistically-significant results (fold change; 2.6 at 15 °C, 2.0 at 10 °C, and 2.0 at 5 °C (Table S3)) relative to 25 °C. Gamma-aminobutyric acid (GABA), fructose, and mannitol show statically significant differences (*p <* 0.05) at 15, 10, and 5 °C. Alpha-ketoglutarate, which serves as an intermediate for several reactions, is regenerated from glutamate in suboptimal temperatures. γ-aminobutyric acid (GABA) shows an increase at suboptimal temperature. In higher plants, GABA is involved in cellular and molecular signaling under stress [48,49]. A very small fraction of free fatty acids is detected, from which 4-acteylbutyric acid (5-oxohexanoate) shows a significant change at 10 °C (3.1) and 5 °C (3.2 fold) as compared to 25 °C. Only a few significant alterations in intermediary fatty acid pool (4-acetylbutyric acid and palmitic acid) are detected (Table S3), most of them remained undetected due to their liability to change on heating. However, 4-Acetylbutyric acid shows a significant increase at suboptimal temperatures whereas arachidic acid shows a decrease at suboptimal temperature. Additionally, linoleic acid, oleic acid, palmitic acid, and stearic acid are lower at 15, 10, and 5 °C, which leads us to speculate that these fatty acids are subjected to elongation and desaturation under suboptimal temperatures.

Many of the significant alterations observed are in amino acids and their derivatives across temperature variants. Determination of total protein contents via % nitrogen shows only subtle variations in total proteins content across temperature treatments (Table 2). Studies have shown that the physiological stress causes protein degradation, which direct cellular metabolism towards substantial adjustments in amino acid pool size by catabolism to meet energy requirements [50]. As a significant increase of N-formyl-L-methionine for temperatures 10 °C and 5 °C is detected, statistical analysis also reveals significant decrease of trans-4-hydroxy-L-proline for those same temperatures. The role of N-formyl-L-Methionine is very crucial in terms of biogenesis of photosystem II by stabilizing the D2 subunits [51]. Microalgae degrades trans-4-hydroxy-L-proline and the product subsequently incorporates into TCA cycle [52]. Overall, metabolites involved in citric acid cycle, amino acid biosynthesis, and carbohydrate metabolism are affected by temperature stress. Amino acids derived from carbohydrate metabolism, such as pyruvate, glycerol-3-phosphate and phosphoenolpyruvate, were elevated in response in response to cold temperatures. There was a significant increase in aromatic amino acids (phenylalanine and tyrosine) and glutamine, while proline decreased significantly during cold stress (Figure 6). Amino acids derived from oxaloacetate (aspartate, asparagine, lysine, threonine, and isoleucine) showed fluctuations between temperature treatments. Generally, these results set an understanding of clear induction of pathway found in photosynthetic organisms, which use recruitment of mono- and polysaccharides into soluble sugars that helps in restructuring of central carbohydrate metabolism [53]. Glutathione (GSH) has a decreased reservoir in the cell under reduced temperature stress. GSH has antioxidant abilities to protect cell against reactive oxygen species (ROS) [54,55], which indicates that cellular metabolite flux is more towards central carbohydrate metabolism, protein degradation, and lipid biosynthesis.

**Figure 7:**
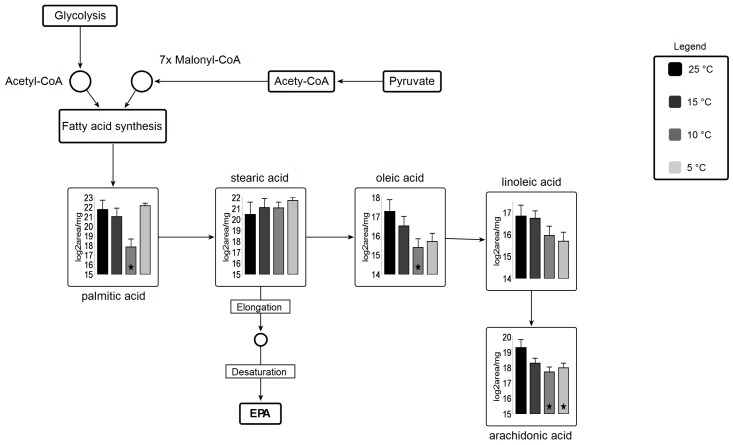
Metabolic pathway for fatty acid biosynthesis coupled with carbohydrate metabolism Circles represent undetected metabolites, averaged log_2_ transformed of biological triplicates (*n* = 9) is shown in the graphs, VANTED software used to perform *t*-test to detect significant changes in metabolomics data. ★ represents statistically significant difference as compared to 25 °C (*p* ≤ 0.05).

Fatty acid biosynthesis is shifted under suboptimal temperature stress (Figure 7). A significant decrease in palmitic acid and oleic acid and arachidonic acid at 10 °C are observed. Lipid analysis indicates significant incorporation of these three fatty acids in all lipid classes, concluding that these fatty acids might be readily taken up by other lipid pools (i.e., membrane lipids) for elongation and desaturation. Stearic acid is a precursor for EPA molecules after being subjected to elongation and desaturation in membranes. Several desaturase enzymes are involved in the biosynthesis of EPA using these fatty acids as substrate [56].

Among amino acids, proline is 0.2 fold lower in the 5 °C culture relative to the 25 °C culture, whereas leucine shows an increase at 10 °C and 5 °C (2 and 1.5 fold, respectively) relative to 25 °C. Studies have shown that leucine biosynthesis is directly involved with lipid biosynthesis regulation and our findings correlate with previous findings [57]. Other amino acids remained statistically unchanged with subtle alterations in amino acid metabolic profile. Valine was the only amino acid residue, which remained unchanged across all temperature variants (Table S3, amino acids). Amino acid derivatives, which showed statistically significant changes at all temperature treatments, include N-formyl-L-methionine (3.0 fold at 5 °C) and trans-4-hydroxy-L-proline (0.2 fold at 5 °C). Bimodal alterations are perceived in polyamines (spermidine) but no statistically significant changes are observed (Table S3, polyamines). A decrease in spermine is observed in suboptimal temperatures as compared to control temperature, while the pool size of putrescine remains unchanged for temperature treatments vs. control.

## 3. Materials and Methods

### 3.1. Culturing of *Nannochloropsis salina*

*Nannochloropsis salina* (CCMP1776) was obtained from the Provasoli-Guillard Centre for the Culture of Marine Phytoplankton (Bigelow, ME, USA) and grown in F/2 medium supplemented with vitamins (UTEX) shaken at 80 rpm and continuous light intensity of 250 μmol m^−2^·s^−1^. After assuring the strain identity (Table S1), six independent environmentally controlled photo-bioreactors (Phenometrics ePBRs 101) were inoculated under the following conditions: an initial optical density 0.45 (λ = 750 nm) in 500 mL F/2 media modified from UTEX with 2X nitrate and 2X phosphate concentrations in a polycarbonate cylindrical reactor vessel, 16:8 h day:night period, 250 μmol m^−2^·s^−1^ light intensity, sparged with 1% CO_2_, 300 rpm magnetic stirring and an initial temperature of 25 °C. After a two-day acclimation and growth period at 25 °C, the ePBRs were diluted to a targeted OD of 0.5 to minimize self-shading and switched to operate under the four different temperature treatments. The experiments were repeated twice to obtain triplicates of four temperature treatments in six ePBRs. Each reactor was randomly assigned to one of the four temperature treatments with optimal temperature (i.e., 25 ± 0.5 °C) as a control and three suboptimal temperatures (i.e., 15 ± 0.1 °C, 10 ± 0.3 °C, and 5 ± 0.7 °C). During each experiment, we duplicated two individual temperature treatments. Cultures were under cold stress for 96 h after two days of acclimation. 2 mL of sample was collected every 24 h to check for OD measurements and cell count. After harvesting at 96 h, the biomass was quenched in liquid nitrogen immediately. Samples were lyophilized using Labconco Freeze Dry System and stored at −80 °C until further analysis.

### 3.2. OD Measurements

Cell density was measured at each harvesting time using DU 530 Life Sciences UV–vis spectrophotometer (Beckman Coulter, Brea, CA USA) (life sciences) at λ 750, 680, and 450 nm (as a measure of cell density, chlorophyll absorbance, and carotenoids absorbance respectively). In addition to these measurements, ratio of λ 750 nm/680 nm, λ 680 nm/450 nm, and λ 750 nm/450 nm were also measured as a factor of monitoring chlorophyll *a* dependent change in culture color and condition.

Cell count was determined by Petroff-Hausser counter using 100× magnification on Zeiss Axioplan Microscope (Zeiss, Germany). 2 μL culture volume was mounted on the counting chamber and cell count for each sample was performed in triplicates. Cells with larger size than *Nannochloropsis salina* and with no chlorophyll content were not included in total cell count. Total cell count was calculated using Equation (1) with the actual values of length, width, and diameter for counting chamber used [58]. Averaged data was collected and analyzed using SAS program (*p* ≤ 0.05).

(1) $$\begin{array}{cc} \;\mathrm{Total}\;\mathrm{cell}\;\mathrm{count} & =\left[\frac{\mathrm{number}\;\mathrm{of}\;\mathrm{cells}}{0.25\;\mathrm{mm}\;L\;\times \;0.25\;\mathrm{mm}\;W\;\times 0.02\;\mathrm{mm}\;D}\right]\;\times \;\left[\frac{1{\;\mathrm{mm}}^{3}}{1\;\mu L}\right]\\ & \times \;\left[\frac{1000\;\mu L}{1\;\mathrm{mL}}\right]\;\times \;{\left[\frac{500\;\mathrm{mL}}{0.5\;L}\right]}^{\;} \end{array}$$

### 3.3. PAM Fluorometery

Photosynthetic measurements were conducted with a Junior-PAM fluorometer (Walz, Germany). Cultures were dark acclimated for 30 min before measuring maximum photochemical quantum yield of photosystem II (Fv/Fm), quantum yield (ΔF/Fm’), and electron transport rate (ETR) of photosystem II (PSII) in photosynthesis, non-photochemical quenching (NPQ), yield of photosystem II, Y(II), and photosynthetic active radiation (PAR). All the measurements were taken at a 24 h interval. Fv/Fm and ETR values were measured over a light intensity program increasing from 0–1500 µmol photons m^−2^·s^−1^. The light from LED sources has very low PAR so that it does not interfere with PAM experimental measurements. Light saturation from PAR occurs at values over 300 µmol photons m^−2^·s^−1^, therefore ETR values for the light intensity program were measured at 280 µmol photons m^−2^·s^−1^.

### 3.4. Dissolved Oxygen Measurements

Dissolved oxygen concentration measurements were conducted using an optical oxygen meter (FireSting) [59]. The oxygen concentration was measured with a 1 mm PMMA fiber directly immersed in the culture at a fixed depth of 13 cm and O_2_ was measured every second for 30 s total. Additionally, temperature was recorded simultaneously with the temperature probe, and these data were used to correct for changes in O_2_ solubility at reduced temperature. Dissolved oxygen measurements (ppm) were corrected for temperature, (25, 15, 10, and 5 °C), atmospheric pressure (640 mmHg) and salinity (3.5%) using dissolved oxygen tables provided by the United States Geological Survey [60].

### 3.5. Elemental Carbon to Nitrogen Ratio Analysis

CHNOS analysis was performed to quantify elemental carbon to nitrogen levels. Approximately 2 mg of lyophilized dried tissue was weighed on a microscale (PerkinElmer AD 6000) and combustion analysis was performed at 975 °C in an oxygen rich environment using organic element analyzer (2400 CHNS/O Series II PerkinElmer, USA). Raw elemental content (%) was used to calculate the C/N ratio and estimate the total protein contents (%*N* × 0.478) [61].

### 3.6. FAME Analysis

Dried samples were subjected to direct transesterification for fatty acid methyl ester (FAME) analysis. For base catalyzed FAME analysis, 50 mg of dried algal biomass was used to extract lipids in 600 μL of chloroform:methanol (2:1) in a glass vial and 2 mL of 2 N KOH in methanol was added and vortexed for 30 s. The mixture was incubated at 50 °C in an HP50 oven shaker (Apollo, San Diego, CA, USA) with continuous shaking at 100 rpm for 30 min. The reaction was quenched by adding 500 μL of 1 M acetic acid followed by vortexing. Total FAMEs were back-extracted into hexane (1 mL aliquot) containing methyl tricosonate, C23:0 as an internal standard at 50 μg/mL. FAME analysis was performed on the samples collected after 96 h growth period with a focus to target PUFAs (C14:n-C20:5n3).

FAMEs were quantified and analyzed by gas chromatography mass spectrometry (GC-MS), using a system composed of a 3800 gas chromatograph (Varian, Palo Alto, CA, USA) and Saturn 2000 R mass spectrometer (Varian, Palo Alto, CA, USA). FAMEs were chromatographically separated on a 60 m × 0.25 mm DB-23 capillary column with a film thickness of 0.25 μm. Helium gas was used a carrier gas at flow rate of 1.0 mL/min. Injector temperature was held at 250 °C. A sample injection of 2 μL was performed. The oven temperature was programmed using the following settings; 100 °C was held for 1 min, ramping at 25 °C/min up to 200 °C, held at 200 °C for 1 min, from 200 °C to 250 °C at the rate of 3 °C/min with a hold of 7 min. Mass spectra were acquired from m/z 50–500. For external calibration and quantification of individual FAMEs, a 1:1 serial dilution series from stock 1 mg mL^−1^ Supelco© 37-Component FAME Mix (Sigma-Aldrich, Saint Louis, MO, USA) was used.

### 3.7. Lipidomics

Total lipids were extracted following the Folch method of lipid extraction with slight modifications [62]. A total of 20–25 mg of lyophilized tissue was used to perform the extraction in tripilcate. Extracts were further diluted in 250 μL of choloroform:methanol:isoporopanol (1:2:4 v/v/v). Diluted lipids were supplemented with 5 μL of 1 M sodium acetate for positive electrospray ionization (ESI) and with 1 M ammonium hydroxide for negative ESI. Analysis of intact lipids was performed using a hybrid linear ion trap Fourier transform ion cyclotron resonance mass spectrometer (Thermo Finnigan, San Jose, CA, USA) equipped with an Advion Triversa Nanomate. Elemental compositions were assigned based upon a series of lipid-specific elemental constraints using PetroOrg software. A signal-to-noise threshold of 10 was selected for peak-picking. Data were processed using in-house developed macros that align component signals across multiple samples and perform library matching based upon a user generated lipid library derived from LIPID MAPS [63,64]. Lipid assignment was performed based on mass and heteroatom class (Table S4). Neutral lipids, galactolipids, and betaine lipids were identified from +ESI data, and sulfo- and phospho- lipids were identified from -ESI data.

### 3.8. Gene Expression Analysis of Four Kennedy Pathway Genes

Genes involved in Kennedy pathway (glycerol-3-phosphate acyltransferase—GPAT, lysophosphatidylcholine acyltransferase—LPAT, phosphatidic acid phosphatase—PAP, diacylglycerol acyltransferase—DGAT) were analyzed using quantitative polymerase chain reaction (qPCR). Total RNA was extracted using PowerBiofilm RNA extraction kit (Mobio, Germantown, MD, USA) followed by cDNA synthesis of 1 μg of RNA using BioRad cDNA synthesis kit. Synthesized cDNA was further diluted to a 1:20 ratio in diethyl pyrocabonate (DEPC) treated water for subsequent qPCR reactions. The reaction was carried out using BioRad SYBR Green Mix. A list of primers and temperature profile is available in supplementary material (Table S1). Gene expression data of GPAT, LPAT, PAP, and DGAT after a 96-h growth period was analyzed after normalization with the housekeeping gene β-actin [65]. Relative fold gene expression (2^−ΔΔ*C*t^) was calculated from the data within each treatment along with standard error of differences.

### 3.9. Metabolomics

Metabolome extraction was carried out in triplicate with 5–10 mg of lyophilized tissue extracted into approximately 1.3 mL methanol:chloroform:water (10:3:1) [66]. The extraction buffer was spiked with 0.32 μM ribitol as an internal standard. Extracts were concentrated to dryness for derivatization and analysis using a vacufuge (Eppendorf, Hauppauge, NY, USA). Derivatization was performed according to Lee and Fiehn [66].

Extracts were analyzed with a GC-TOF-MS system composed of a 7890 A GC System (Agilent Technologies, Santa Clara, CA, USA) and a LECO Pegasus HT High Throughput TOF-MS (Leco, Saint Joseph, MI, USA). Spectral deconvolution and identification were performed using ChromaTOF 4.41 and the Fiehn metabolomics library [67]. Peak integration and alignment for all samples was performed in MET-IDEA V2.08, using ribitol as the retention time locker. Peak areas were normalized to the ISTD ribitol and tissue weight then log_2_ transformed. These values were used to perform statistical analyses using MetaboAnalyst 4.0 [68]. VANTED (version 2.6.4, Monash University, Melbourne, Australia) [69] software was then used for mapping individual metabolite data onto carbohydrate, amino acid, and fatty acid pathway networks. Pathway maps were designed based on KEGG pathways representing primary metabolic pathways observed in the model alga *Chlamydomonas reinhardtii*.

### 3.10. Statistical Analysis

Statistical analysis with *p* ≤ 0.05 was performed using SAS version 10.0 software (SAS institute, Cary, NC, USA). A randomized complete block design was used to study the effect of temperature treatment in two blocks on different parameters. Block was treated as a fixed measurement and interaction of days and temperature treatment was evaluated for OD measurement, Fv/Fm, ETR values, cell count, and %saturation of oxygen. Two-way repeated measured ANOVA with post-hoc analysis (Fisher’s LSD test) was used to see the significance of two factors, temperature and growth period on OD, Fv/Fm, cell count, and % saturation of oxygen. The effects of suboptimal temperature treatments were studied for fatty acid profile, lipidomics, gene expression studies and metabolomics after 96 h growth period using ANOVA with post hoc analysis. The covariance structure for each parameter was checked and raw means and standard error values are presented in result analysis section. We represented letters only on those variables for which F-test was significant (*p <* 0.05).

## 4. Conclusions

*Nannochloropsis salina* is a promising candidate strain for sustainable production of high value co-products including long chain polyunsaturated fatty acids (PUFAs), omega-3 fatty acids, omega-6 fatty acids, and EPA. Cold stress instigates at 15 °C after 48 h of experimental conditions, where photosynthetic measurements (Fv/Fm values) were significantly decreased (*p <* 0.05) compared to optimal temperature treatment. After 96 h, cultures grown at 15 °C were acclimated to cold stress and had adjusted photosynthetic activity. FAME analysis of *N. salina* cultivated under cold stress showed a 2-fold increase in PUFAs at 15 °C and 10 °C, suggesting that these temperature treatments are more suitable for PUFAs production. These studies have found that *N. salina* under suboptimal conditions (15 °C) produced higher TAGs, with EPA at all three (*sn*) positions. Gene expression results also revealed that enzymes responsible for Kennedy pathway at ER membranes showed changes in expression at reduced temperature treatment. A non-significant increase in the expression of genes involved in Kennedy pathway was observed for this study suggesting that transcriptional fluctuations do occur under suboptimal growth conditions to adjust cellular machinery in response to physiological stress. High resolution mass spectral lipid analysis FT-ICR data provided detailed insight into lipid remodeling in *N. salina* at reduced temperature, specifically with respect to lipid remodeling of galactolipids (MGDG, DGDG), betaine lipids (MGTS, DGTS), and TAGs. Also, we have observed highly polyunsaturated lipid species in TAGs along with assimilation of EPA into *sn2*, *sn3* position of TAG molecule. Metabolite profiles illustrate the flux transition of central carbohydrate metabolites under constant reduced temperature stress. We observed fluctuations central carbohydrate metabolism, amino acid biosynthesis, and fatty acid biosynthesis. However, additional experiments are required to investigate the flux of lipid pool under reduced temperature treatment along with the study of lipid remodeling pathways involved in TAG production.

## Figures and Tables

**Table 1:** Physiological parameters study in *N. salina* under cold stress. Optical density measurements at 750 nm indicating cell density, were used to determine growth over the 96 h growth period, total cell count was monitored using counting chamber. Fv/Fm ratio and ETR values (at 285 µmol photons m^−2^·s^−1^) are listed for a 96 h time period. % Saturation of oxygen was calculated by normalizing dissolved oxygen (DO) values (ppm) with temperature, salinity and atmospheric pressure. All data points show average of triplicate along with standard errors. Statistical analysis was performed using SAS, where A, B, C, D represents statistically significant mean separation for differences between temperature on each day at *p* ≤ 0.05.

Optical Density Measurements (750 nm)								
Temp/H	25 °C	Post-Hoc	15 °C	Post-Hoc	10 °C	Post-Hoc	5 °C	Post-Hoc
0	0.46 ± 0.01	A	0.45 ± 0.01	A	0.44 ± 0.01	A	0.46 ± 0.01	A
24	0.51 ± 0.01	A	0.51 ± 0.00	AB	0.48 ± 0.03	B	0.49 ± 0.02	AB
48	0.53 ± 0.01	A	0.52 ± 0.02	AB	0.49 ± 0.02	C	0.47 ± 0.02	BC
72	0.57 ± 0.01	A	0.52 ± 0.02	B	0.50 ± 0.02	B	0.48 ± 0.02	B
96	0.60 ± 0.01	A	0.50 ± 0.01	B	0.51 ± 0.01	B	0.49 ± 0.02	B
Total Cell count (per mL)								
0	6.1 × 10^9^ ± 3.9 × 10^8^	A	5.1 × 10^9^ ± 2.5 × 10^8^	A	4.8 × 10^9^ ± 1.0 × 10^7^	A	4.7 × 10^9^ ± 2.4 × 10^8^	A
24	7.3 × 10^9^ ± 5.5 × 10^8^	A	6.3 × 10^9^ ± 2.7 × 10^8^	A	5.6 × 10^9^ ± 5.1 × 10^7^	A	5.5 × 10^9^ ± 1.3 × 10^8^	A
48	9.3 × 10^9^ ± 4.0 × 10^8^	A	7.2 × 10^9^ ± 2.4 × 10^8^	B	6.9 × 10^9^ ± 2.4 × 10^8^	B	6.1 × 10^9^ ± 6.8 × 10^7^	B
72	1.1 × 10^10^ ± 4.3 × 10^8^	A	9.5 × 10^9^ ± 1.1 × 10^8^	A	7.6 × 10^9^ ± 9.3 × 10^7^	B	7.3 × 10^9^ ± 2.2 × 10^8^	B
96	1.4 × 10^10^ ± 5.1 × 10^8^	A	1.2 × 10^10^ ± 5.1 × 10^8^	B	9.5 × 10^9^ ± 1.4 × 10^8^	C	7.8 × 10^9^ ± 2.9 × 10^8^	C
Photosynthetic Activity (Fv/Fm)								
0	0.55 ± 0.02	A	0.55 ± 0.00	A	0.55 ± 0.01	A	0.54 ± 0.01	A
24	0.56 ± 0.00	A	0.55 ± 0.02	AB	0.55 ± 0.03	AB	0.48 ± 0.05	B
48	0.57 ± 0.01	A	0.54 ± 0.04	B	0.52 ± 0.05	BC	0.39 ± 0.10	C
72	0.57 ± 0.01	A	0.57 ± 0.01	A	0.56 ± 0.02	A	0.53 ± 0.02	A
96	0.56 ± 0.00	A	0.50 ± 0.01	A	0.39 ± 0.05	B	0.28 ± 0.07	C
ETR Values (µmol photons m^−2^·s^−1^)								
0	17.00 ± 3.23	A	14.13 ± 3.13	A	16.20 ± 2.33	A	17.83 ± 3.52	A
24	19.00 ± 1.61	A	11.47 ± 3.58	A	9.27 ± 2.22	B	8.87 ± 3.72	AB
48	15.30 ± 4.38	A	11.13 ± 2.45	A	11.07 ± 3.41	B	7.90 ± 1.80	AB
72	20.47 ± 4.72	A	12.77 ± 1.44	A	11.10 ± 2.57	AB	6.50 ± 1.33	B
96	15.80 ± 6.06	A	8.70 ± 1.55	A	8.37 ± 1.73	B	5.50 ± 1.40	B
% Saturation of Oxygen								
0	119.04 ± 3.41	A	86.37 ± 7.79	B	63.88 ± 4.86	C	70.76 ± 5.53	C
24	91.26 ± 3.66	A	88.99 ± 2.02	A	86.75 ± 4.68	AB	74.46 ± 5.34	B
48	89.51 ± 2.20	A	87.04 ± 3.71	A	86.44 ± 7.63	A	72.39 ± 6.72	B
72	96.43 ± 6.15	A	89.08 ± 2.01	B	88.38 ± 2.86	B	78.69 ± 2.28	B
96	93.29 ± 4.64	A	88.48 ± 2.39	B	89.50 ± 5.27	AB	69.85 ± 5.43	C

**Table 2:** Elemental combustion analysis in *N. salina* during reduced cultivation temperature. Elemental carbon, nitrogen, C/N, and total protein content determination for *Nannochloropsis salina* after a 96-h growth period: Samples were collected in triplicates (*n* = 3) and analyzed using SAS (*p* ≤ 0.05). No significant difference was observed with elemental analysis for temperature treatments. % w/w elemental analysis of carbon, nitrogen, carbon to nitrogen, and calculated total protein content with ±standard error mean (SEM) are listed in table. * Total protein contents (%w/w) were estimated using formula = %N × 4.78.

Growth Temperatures				
% Composition	25 °C	15 °C	10 °C	5 °C
Carbon	41.31 ± 0.76	44.07 ± 0.41	41.88 ± 0.38	40.86 ± 1.54
Nitrogen	5.98 ± 0.06	6.59 ± 0.07	6.00 ± 0.06	5.87 ± 0.15
C/N	6.91 ± 0.09	6.69 ± 0.68	6.98 ± 0.09	6.95 ± 0.15
* Total protein	28.57 ± 0.30	31.49 ± 0.34	28.70 ± 0.29	28.05 ± 0.70

**Table 3:** FAME analysis: FAMEs detected in *N. salina* dried biomass (mg/g). Percentages by dried cell weight are provided for total fatty acid (TFA) and EPA content. Totals for saturated fatty acids (SFA), unsaturated fatty acids (UFA), monounsaturated fatty acids (MUFA) and PUFA are shown, and proportions of SFA/TFA, UFA/TFA, PUFA/TFA, PUFA/UFA, and MUFA/PUFA were calculated as well. Averaged values and ±SEM are provided (*n* = 3) where ^a–d^ represents statistically significant mean separations of differences at *p* ≤ 0.05.

Growth Temperatures				
Fatty Acid (mg/g)	25 °C	15 °C	10 °C	5 °C
C14:0	9.12 ± 0.59	7.62 ± 1.31	8.34 ± 0.38	9.80 ± 0.10
C14:1	2.03 ± 1.49	0.5 ± 0.19	1.90 ± 1.51	5.35 ± 0.10
C16:0	42.94 ± 3.55	33.42 ± 6.88	37.28 ± 3.07	43.71 ± 1.05
C16:1	43.4 ± 2.13	42.85 ± 4.80	45.66 ± 0.62	51.26 ± 0.76
C16:2	0.95 ± 0.10	0.83 ± 0.16	0.72 ± 0.09	0.92 ± 0.10
C16:3	0.14 ± 0.06	0.07 ± 0.05	0.13 ± 0.06	0.07 ± 0.05
C18:0	5.93 ± 1.45	3.66 ± 2.30	4.16 ± 1.20	6.33 ± 1.09
C18:1n9t	0.94 ± 0.34	0.93 ± 0.12	1.80 ± 0.25	1.06 ± 0.48
C18:1n9c	1.95 ± 0.70	1.89 ± 0.23	3.59 ± 0.44	2.72 ± 0.86
C18:2n6t	0.75 ± 0.07	0.65 ± 0.07	0.97 ± 0.12	0.87 ± 0.12
C18:2n6c	0.11 ± 0.03	0.08 ± 0.02	0.08 ± 0.02	0.08 ± 0.02
C18:3n6	1.19 ± 0.13	0.99 ± 0.50	0.46 ± 0.46	2.14 ± 0.5
C18:3n3	0.07 ± 0.02	0.06 ± 0.02	0.08 ± 0.01	0.10 ± 0.01
C20:3n6	0.80 ^a^ ± 0.09	1.60 ^a^ ± 0.34	2.77 ^b^ ± 0.06	3.01 ^b^ ± 0.31
C20:4n6	0.00 ± 0.00	1.45 ± 1.45	5.46 ± 2.74	4.45 ± 4.45
C20:3n3	0.00 ^a^ ± 0.00	0.01 ^b^ ± 0.01	0.02 ^c^ ± 0.01	0.02 ^c^ ± 0.01
C20:5n3	77.45 ± 5.77	73.14 ± 12.71	75.9 ± 6.4	91.26 ± 3.81
% TFA (w/w)	18.78 ± 1.43	16.98 ± 2.75	18.93 ± 1.17	22.32 ± 0.31
% EPA (w/w)	7.74 ± 0.58	7.31 ± 1.27	7.59 ± 0.64	9.13 ± 0.38
∑ SFA	57.98 ± 5.53	44.71 ± 10.44	49.78 ± 4.64	59.84 ± 1.98
∑ UFA	129.78 ± 8.80	125.05 ± 17.13	139.54 ± 7.08	163.31 ± 4.02
∑ MUFA	48.31 ± 2.90	46.18 ± 4.73	52.94 ± 2.78	60.38 ± 2.14
∑ PUFA	81.46 ± 5.90	78.87 ± 12.51	86.59 ± 4.30	102.93 ± 1.90
∑ SFA/TFA	0.31 ^a^ ± 0.01	0.26 ^b^ ± 0.02	0.26 ^b^ ± 0.01	0.27 ^b^ ± 0.01
∑ UFA/TFA	0.69 ^a^ ± 0.01	0.74 ^b^ ± 0.02	0.74 ^b^ ± 0.01	0.73 ^b^ ± 0.01
∑ PUFA/TFA	0.43 ^a^ ± 0.00	0.47 ^b^ ± 0.00	0.46 ^b^ ± 0.01	0.46 ^b^ ± 0.00
∑ PUFA/UFA	0.63 ± 0.00	0.63 ± 0.01	0.62 ± 0.00	0.63 ± 0.00
∑ MUFA/PUFA	1.17 ± 0.07	1.07 ± 0.10	1.32 ± 0.04	1.49 ± 0.13

**Table 4:** Fold-changes in primary lipid pools: Variation in distribution of major lipid classes among different temperature was studied using FT-ICR mass spectrometry. Heat map of total lipid pool in *N. salina* at suboptimal temperatures (15, 10, and 5 °C) were calculated by adding S/N (threshold ≥ 10) values of individual species within each lipid class, and normalized to dry biomass. Fold change data was generated by calculating treatment vs. control ratio. Average values calculated from triplicates (*n* = 3).

Lipid Class	25 °C	15 °C	10 °C	5 °C
**∑ MGTS**				
**∑ DGTS**				
**∑ DAG**				
**∑ TAG**				
**∑ MGDG**				
**∑ DGDG**				
**∑ SQDG**				
**∑ PG**				

∎: 0.5; ∎: 0.75; □: 1.0; ∎: 1.25; ∎: 1.5; ∎: 2.0.

## Supplementary Materials

The following are available online at http://www.mdpi.com/1660-3397/16/11/425/s1, Table S1: List of primer sequences used in analysis; Table S2: Growth rate and doubling time measurements for *N. salina* under suboptimal temperature treatment; Table S3: Fold changes in selected metabolites under cold stress; Table S4: Classification of lipid based of their *m*/*z*.
