# Supplementary material for: Suboptimal Temperature Acclimation Affects Kennedy Pathway Gene Expression, Lipidome and Metabolite Profile of Nannochloropsis salina during PUFA Enriched TAG Synthesis
Source: Mar Drugs. 2018 Nov 1;16(11):425. doi: 10.3390/md16110425 (PMC6266265; doi:10.3390/md16110425)
Supplement: Supplementary file 1 [file marinedrugs-16-00425-s001.pdf]

marine drugs

Supplementary material

Strain identification

To confirm strain identity, total genomic DNA was extracted using PowerPlantDNA extraction kit (Mo BIO). ITS regions were amplified using two sets of primers (Table S1). We used 95 °C denaturation temperature for 1 min, 59.5 °C annealing temperature for 30 s, and 72 °C extension temperature for 30 s in thermocycler (eppendorf, Mastercycler *gradient*). Amplicons were sent for sequencing to Functional Biosciences™. The obtained sequences were analyzed using Geneious and BLAST tools.

Table S1. List of primer sequences used in analysis.

Primer Name	Sequence	Tm (°C)
ITS 1-F	GTTTGCACGCCAAGGTAATG	59.5
ITS 1-R	CCACCACCCATAGAATCAAGAA	59.5
ITS-2-F	GATACGACCTTGGTGGTCTAT TT	59.5
ITS-2-R	GAGCTGATGACTCGCGTTTA	59.5
GPAT-F	GTG TCTTCGCTTGTCATCCG	60
GPAT-R	GGCGTCTGCATTGGATAGGT	60
PAP3-F	CCTCCGAAAACCATGCTTCC	60
PAP3-R	CTTCCTACTGCCGGTGGTTC	60
LPAT-F	TGTACGACGCTCCCTTCAAC	60
LPAT-R	CTCCGACATGGCAAAAGTGC	60
DGAT2-F	CCATGTGCTCACAGGTGG	60
DGAT2-R	AGTATAGCCGTGCTGTAACGC	60
actinN4-F	GTGCGGGACATCAAGGAGAA	60
actinN4-R	ATCACGTTACCATCCGGCAG	60

For qPCR analysis, we used CFX Connect Real time System with the following settings: 95 °C denaturation temperature for 5 min, 95 °C for 15 seconds, 58 °C for 15 s, 72 °C for 30 s (35 cycle), Melt curve: 50 °C to 95 °C with the increment of 0.5 °C after every 5 s.

Table S2: Growth rate and doubling time measurements for *N. salina* under suboptimal temperature treatment

Growth rate and Doubling time measurements (day)				
Temp/Hr	25 °C	15 °C	10 °C	5 °C
Growth Rate	0.08 ± 0.01	0.06 ± 0.01	0.05 ± 0.01	0.04 ± 0.01
Doubling time	8.55 ± 0.65	11.11 ± 1.32	13.44 ± 1.75	16.97 ± 2.31

Table S3. Fold changes in selected metabolites under cold stress. Metabolites were identified from Fiehn library using Fiehn retention index and calculated retention index (Fiehn RI-Calc. RI ≤ ±2000). **Bold** values represent statistically significant difference of metabolite pool at given temperature compared to control (t-test; $p < 0.05$).

ompound Name	Fiehn RI	Calculated RI	25 °C	15 °C	10 °C	5 °C
Sugar class						
D-ARABITOL	574079	573886	1.00	0.84	0.28	0.27
FRUCTOSE	577729	577514	1.00	1.01	2.15	2.45
GALACTOSE	648681	648161	1.00	1.30	1.03	1.16
GLUCOSE	650947	650697	1.00	0.49	0.76	0.55
MANNITOL	665262	664446	1.00	0.48	0.18	0.17
MANNOSE	646178	645224	1.00	0.10	0.14	0.12
MYO-INOSITOL	729867	730189	1.00	0.44	0.06	0.03
SORBOSE	639323	637615	1.00	0.44	1.00	0.69
SUCROSE	914209	913454	1.00	0.62	0.80	0.63
TREHALOSE	947579	947874	1.00	0.96	1.92	1.63
XYLOSE	542483	541638	1.00	1.34	1.39	1.48
Sugar phosphates						
FRUCTOSE 1,6-BISPHOSPHATE	935649	933916	1.00	0.43	0.39	0.27
FRUCTOSE 2,6-BISPHOSPHATE	765677	764663	1.00	0.66	0.78	0.87
GLYCEROL 1-PHOSPHATE	575744	575633	1.00	1.15	1.46	0.98
PYRIDOXAL PHOSPHATE	792621	792275	1.00	0.88	1.03	1.22
Organic acids						
2-KETOGLUTARIC ACID	507334	507778	1.00	2.63	2.01	1.95
ASPARTIC ACID	479202	479711	1.00	0.72	0.64	0.25
CITRIC ACID	617832	618127	1.00	0.68	1.30	1.36
GAMMA-AMINOBUTYRIC ACID	487696	488026	1.00	1.49	1.95	2.07
GENTISIC ACID	602031	603977	1.00	0.88	0.98	0.99
GLUCOSE	650947	650830	1.00	0.49	0.76	0.55
GLUCURONIC ACID	667638	667249	1.00	0.20	0.14	0.16
GLUTARIC ACID	420308	419504	1.00	0.33	0.67	0.47
MALEIC ACID	365916	366250	1.00	0.56	0.48	0.40
MALIC ACID	462908	463620	1.00	0.54	0.53	0.81
MALONIC ACID	430110	430366	1.00	1.17	1.46	1.15
METHYLMALONIC ACID	311544	312137	1.00	0.88	0.87	0.55
SHIKIMIC ACID	612089	612787	1.00	0.86	1.98	0.82
Fatty acids						
4-ACETYLBUTYRIC ACID	385939	384238	1.00	2.05	3.16	3.20
ARACHIDIC ACID	855981	856178	1.00	0.21	0.42	0.33
ELAIDIC ACID	781663	781904	1.00	0.36	0.16	0.19
LAURIC ACID	547162	547819	1.00	0.44	0.38	0.62
LIGNOCERIC ACID	977654	977822	1.00	0.37	0.18	0.23
LINOLEIC ACID	777515	777459	1.00	0.70	0.45	0.38
OLEIC ACID	780313	779479	1.00	0.36	0.16	0.19
PALMITIC ACID	714610	715262	1.00	0.33	0.08	0.34
STEARIC ACID	787954	788100	1.00	0.58	0.40	0.53
Organic compounds						
GLUTATHIONE	908197	908711	1.00	0.56	0.49	0.34
SALICYLALDEHYDE	406850	405157	1.00	1.40	2.45	2.26
AMIDES						
ADIPAMIDE	543404	542444	1.00	1.21	2.14	2.17
N-OLEOYLDOPAMINE	971460	972808	1.00	0.14	0.24	0.15
NORVALINE	327136	328921	1.00	1.33	1.54	1.69
Amino acids derivatives						
N-FORMYL-L-METHIONINE	566834	566899	1.00	0.85	1.99	3.01
TRANS-4-HYDROXY-L-PROLINE	483802	484673	1.00	0.33	0.24	0.20
Amino acids						
ALANINE	398918	399123	1.00	2.31	2.79	2.86
ASPARAGINE	476536	476761	1.00	1.27	0.13	0.43

CITRULLINE	621977	622265	1.00	0.25	0.98	0.88
GLUTAMIC ACID	528609	529277	1.00	0.42	0.10	0.03
GLUTAMINE	600736	600507	1.00	1.52	1.91	1.61
ISOLEUCINE	293322	293472	1.00	1.16	0.74	0.86
LEUCINE	346389	347854	1.00	0.94	2.01	1.50
PROLINE	363983	364639	1.00	0.51	0.35	0.23
THREONINE	361557	361819	1.00	0.93	1.18	1.21
TYROSINE	671841	671567	1.00	0.91	1.02	1.06
SERINE	395017	396441	1.00	0.73	0.85	0.62
VALINE	239669	238687	1.00	0.94	0.97	1.07
Poly amines						
SPERMIDINE	793225	792275	1.00	2.21	1.08	1.69
SPERMINE	952692	954650	1.00	0.76	0.46	0.50
PUTRESCINE	588298	587292	1.00	1.11	0.96	1.38
Saccharides						
CELLOBIOSE	933726	933374	1.00	1.08	1.66	1.84
GENTIOBIOSE	967297	967659	1.00	0.10	0.27	0.23
Others						
ETHANOLAMINE	344667	345840	1.00	2.66	4.73	5.22
KETOVALERIC ACID	268884	270645	1.00	1.09	0.33	0.41

Table S4. Classification of lipids based of their m/z.

Molecular Formula	Heteroatom Class	m/z	DBE	Carbon	sp
**MGTS [Acyl C= C-10], [Acyl DBE= DBE-2]**					
$C_{26}H_{51}N_1O_6Na_1$	$N_1O_6Na_1$	496.36096	2	26	[16:0]
$C_{26}H_{49}N_1O_6Na_1$	$N_1O_6Na_1$	494.34540	3	26	[16:1]
$C_{30}H_{51}N_1O_6Na_1$	$N_1O_6Na_1$	544.36087	6	30	[20:4]
$C_{30}H_{49}N_1O_6Na_1$	$N_1O_6Na_1$	542.34516	7	30	[20:5]
**DGTS [Acyl C= C-10], [Acyl DBE= DBE-3]**					
$C_{40}H_{75}N_1O_7Na_1$	$N_1O_7Na_1$	704.54322	4	40	[30:1]
$C_{40}H_{73}N_1O_7Na_1$	$N_1O_7Na_1$	702.52759	5	40	[30:2]
$C_{42}H_{79}N_1O_7Na_1$	$N_1O_7Na_1$	732.57424	4	42	[32:1]
$C_{42}H_{77}N_1O_7Na_1$	$N_1O_7Na_1$	730.55926	5	42	[32:2]
$C_{42}H_{75}N_1O_7Na_1$	$N_1O_7Na_1$	728.54328	6	42	[32:3]
$C_{44}H_{83}N_1O_7Na_1$	$N_1O_7Na_1$	760.60548	4	44	[34:1]
$C_{44}H_{81}N_1O_7Na_1$	$N_1O_7Na_1$	758.59035	5	44	[34:2]
$C_{44}H_{79}N_1O_7Na_1$	$N_1O_7Na_1$	756.57461	6	44	[34:3]
$C_{44}H_{77}N_1O_7Na_1$	$N_1O_7Na_1$	754.55921	7	44	[34:4]
$C_{44}H_{75}N_1O_7Na_1$	$N_1O_7Na_1$	752.54337	8	44	[34:5]
$C_{46}H_{81}N_1O_7Na_1$	$N_1O_7Na_1$	782.59005	7	46	[36:4]
$C_{46}H_{79}N_1O_7Na_1$	$N_1O_7Na_1$	780.57496	8	46	[36:5]
$C_{46}H_{77}N_1O_7Na_1$	$N_1O_7Na_1$	778.55921	9	46	[36:6]
$C_{46}H_{75}N_1O_7Na_1$	$N_1O_7Na_1$	776.54381	10	46	[36:7]
$C_{50}H_{81}N_1O_7Na_1$	$N_1O_7Na_1$	830.59011	11	50	[40:8]
$C_{50}H_{79}N_1O_7Na_1$	$N_1O_7Na_1$	828.57412	12	50	[40:9]
$C_{50}H_{77}N_1O_7Na_1$	$N_1O_7Na_1$	826.55756	13	50	[40:10]
**DAG [Acyl C= C-3], [Acyl DBE= DBE-2]**					
$C_{35}H_{66}O_5Na_1$	O_5Na_1	589.48057	3	35	[32:1]
$C_{35}H_{64}O_5Na_1$	O_5Na_1	587.46482	4	35	[32:2]
$C_{37}H_{62}O_5Na_1$	O_5Na_1	609.44928	7	37	[34:5]
$C_{39}H_{66}O_5Na_1$	O_5Na_1	637.48016	7	39	[36:5]
$C_{39}H_{64}O_5Na_1$	O_5Na_1	635.46498	8	39	[36:6]
$C_{43}H_{66}O_5Na_1$	O_5Na_1	685.48010	11	43	[40:9]
$C_{43}H_{64}O_5Na_1$	O_5Na_1	683.46439	12	43	[40:10]
**TAG [Acyl C= C-3], [Acyl DBE= DBE-3]**					
$C_{47}H_{86}O_6Na_1$	O_6Na_1	769.63154	5	47	[44:2]
$C_{49}H_{92}O_6Na_1$	O_6Na_1	799.67861	4	49	[46:1]
$C_{49}H_{90}O_6Na_1$	O_6Na_1	797.66329	5	49	[46:2]
$C_{49}H_{88}O_6Na_1$	O_6Na_1	795.64755	6	49	[46:3]
$C_{51}H_{96}O_6Na_1$	O_6Na_1	827.71046	4	51	[48:1]
$C_{51}H_{94}O_6Na_1$	O_6Na_1	825.69396	5	51	[48:2]
$C_{51}H_{92}O_6Na_1$	O_6Na_1	823.67905	6	51	[48:3]
$C_{51}H_{90}O_6Na_1$	O_6Na_1	821.66271	7	51	[48:4]
$C_{51}H_{88}O_6Na_1$	O_6Na_1	819.64786	8	51	[48:5]
$C_{53}H_{100}O_6Na_1$	O_6Na_1	855.74094	4	53	[50:1]
$C_{53}H_{98}O_6Na_1$	O_6Na_1	853.72583	5	53	[50:2]
$C_{53}H_{96}O_6Na_1$	O_6Na_1	851.71032	6	53	[50:3]
$C_{53}H_{94}O_6Na_1$	O_6Na_1	849.69446	7	53	[50:4]
$C_{53}H_{92}O_6Na_1$	O_6Na_1	847.67833	8	53	[50:5]
$C_{53}H_{90}O_6Na_1$	O_6Na_1	845.66351	9	53	[50:6]
$C_{55}H_{102}O_6Na_1$	O_6Na_1	881.75673	5	55	[52:2]
$C_{55}H_{100}O_6Na_1$	O_6Na_1	879.74157	6	55	[52:3]
$C_{55}H_{98}O_6Na_1$	O_6Na_1	877.72628	7	55	[52:4]
$C_{55}H_{96}O_6Na_1$	O_6Na_1	875.70974	8	55	[52:5]
$C_{55}H_{94}O_6Na_1$	O_6Na_1	873.69436	9	55	[52:6]
$C_{55}H_{92}O_6Na_1$	O_6Na_1	871.67904	10	55	[52:7]
$C_{59}H_{94}O_6Na_1$	O_6Na_1	921.69430	13	59	[56:10]
$C_{59}H_{92}O_6Na_1$	O_6Na_1	919.67860	14	59	[56:11]
$C_{63}H_{92}O_6Na_1$	O_6Na_1	967.67931	18	63	[60:15]
**MGDG [Acyl C= C-9], [Acyl DBE= DBE-3]**					
$C_{41}H_{76}O_{10}Na_1$	$O_{10}Na_1$	751.53308	4	41	[32:1]
$C_{41}H_{74}O_{10}Na_1$	$O_{10}Na_1$	749.51720	5	41	[32:2]
$C_{41}H_{68}O_{10}Na_1$	$O_{10}Na_1$	743.47032	8	41	[32:5]
$C_{43}H_{78}O_{10}Na_1$	$O_{10}Na_1$	777.54929	5	43	[34:2]
$C_{43}H_{72}O_{10}Na_1$	$O_{10}Na_1$	771.50108	8	43	[34:5]

$C_{44}H_{68}O_{10}Na_1$	$O_{10}Na_1$	779.47058	11	44	[35:8]
$C_{45}H_{82}O_{10}Na_1$	$O_{10}Na_1$	805.58063	5	45	[36:2]
$C_{45}H_{76}O_{10}Na_1$	$O_{10}Na_1$	799.53328	8	45	[36:5]
$C_{45}H_{74}O_{10}Na_1$	$O_{10}Na_1$	797.51772	9	45	[36:6]
$C_{47}H_{78}O_{10}Na_1$	$O_{10}Na_1$	825.54831	9	47	[38:6]
$C_{47}H_{76}O_{10}Na_1$	$O_{10}Na_1$	823.53309	10	47	[38:7]
$C_{47}H_{74}O_{10}Na_1$	$O_{10}Na_1$	821.51746	11	47	[38:8]
$C_{49}H_{78}O_{10}Na_1$	$O_{10}Na_1$	849.54897	11	49	[40:8]
$C_{49}H_{76}O_{10}Na_1$	$O_{10}Na_1$	847.53245	12	49	[40:9]
$C_{49}H_{74}O_{10}Na_1$	$O_{10}Na_1$	845.51681	13	49	[40:10]
**DGDG [Acyl C= C-15], [Acyl DBE= DBE-4]**					
$C_{47}H_{86}O_{15}Na_1$	$O_{15}Na_1$	913.58644	5	47	[32:1]
$C_{47}H_{84}O_{15}Na_1$	$O_{15}Na_1$	911.57079	6	47	[32:2]
$C_{47}H_{82}O_{15}Na_1$	$O_{15}Na_1$	909.55514	7	47	[32:3]
$C_{49}H_{88}O_{15}Na_1$	$O_{15}Na_1$	939.60209	6	49	[34:2]
$C_{49}H_{86}O_{15}Na_1$	$O_{15}Na_1$	937.58644	7	49	[34:3]
$C_{49}H_{82}O_{15}Na_1$	$O_{15}Na_1$	933.55514	9	49	[34:5]
$C_{51}H_{86}O_{15}Na_1$	$O_{15}Na_1$	961.58644	9	51	[36:5]
$C_{51}H_{84}O_{15}Na_1$	$O_{15}Na_1$	959.57079	10	51	[36:6]
**SQDG [Acyl C= C-9], [Acyl DBE= DBE-3]**					
$C_{41}H_{77}O_{12}S_1$	$O_{12}S_1$	793.51412	3	41	[32:0]
$C_{41}H_{73}O_{12}S_1$	$O_{12}S_1$	789.48282	5	41	[32:2]
$C_{45}H_{75}O_{12}S_1$	$O_{12}S_1$	815.49792	8	45	[36:5]
**PG [Acyl C= C-6], [Acyl DBE= DBE-2]**					
$C_{40}H_{76}O_{10}P_1$	$O_{10}P_1$	747.51816	3	40	[34:1]
$C_{42}H_{72}O_{10}P_1$	$O_{10}P_1$	767.48686	7	42	[36:5]
$C_{42}H_{68}O_{10}P_1$	$O_{10}P_1$	763.45556	9	42	[36:7]
**FFA [Acyl C= C-0], [Acyl DBE= DBE-0]**					
$C_{16}H_{31}O_2$	O_2	255.23240	0	16	[16:0]
$C_{16}H_{29}O_2$	O_2	253.21675	1	16	[16:1]
$C_{18}H_{35}O_2$	O_2	283.26370	0	18	[18:0]
$C_{18}H_{33}O_2$	O_2	281.24805	1	18	[18:1]
$C_{18}H_{31}O_2$	O_2	279.23240	2	18	[18:2]
$C_{20}H_{31}O_2$	O_2	303.23240	4	20	[20:4]
$C_{20}H_{29}O_2$	O_2	301.29500	5	20	[20:5]
